# Transcriptomic and Physiological Analyses for the Role of Hormones and Sugar in Axillary Bud Development of Wild Strawberry Stolon

**DOI:** 10.3390/plants13162241

**Published:** 2024-08-13

**Authors:** Genqian Lan, Mingzhao Wu, Qihang Zhang, Bo Yuan, Guangxin Shi, Ni Zhu, Yibingyue Zheng, Qiang Cao, Qin Qiao, Ticao Zhang

**Affiliations:** 1 School of Agriculture, Yunnan University, Kunming 650091, China; langenqian@sina.com (G.L.); wumingzhao101@outlook.com (M.W.); qhang118155@163.com (Q.Z.); yuebo1255@foxmail.com (B.Y.); S18788391634@163.com (G.S.); 15287889166@163.com (N.Z.); 16600034933@163.com (Y.Z.); 2 College of Horticulture and Landscape, Yunnan Agricultural University, Kunming 650201, China; qiang_cao@outlook.com; 3 Key Laboratory of Phytochemistry and Natural Medicines, Kunming Institute of Botany, Chinese Academy of Sciences, Kunming 650201, China

**Keywords:** *Fragaria nilgerrensis*, strawberry stolon, transcriptome, phytohormone, sugar

## Abstract

Strawberries are mainly propagated by stolons, which can be divided into monopodial and sympodial types. Monopodial stolons consistently produce ramets at each node following the initial single dormant bud, whereas sympodial stolons develop a dormant bud before each ramet. Sympodial stolon encompasses both dormant buds and ramet buds, making it suitable for studying the formation mechanism of different stolon types. In this study, we utilized sympodial stolons from *Fragaria nilgerrensis* as materials and explored the mechanisms underlying sympodial stolon development through transcriptomic and phytohormonal analyses. The transcriptome results unveiled that auxin, cytokinin, and sugars likely act as main regulators. Endogenous hormone analysis revealed that the inactivation of auxin could influence bud dormancy. Exogenous cytokinin application primarily induced dormant buds to develop into secondary stolons, with the proportion of ramet formation being very low, less than 10%. Furthermore, weighted gene co-expression network analysis identified key genes involved in ramet formation, including auxin transport and response genes, the cytokinin activation gene *LOG1*, and glucose transport genes *SWEET1* and *SFP2*. Consistently, in vitro cultivation experiments confirmed that glucose enhances the transition of dormant buds into ramets within two days. Collectively, cytokinin and glucose act as dormant breakers, with cytokinin mainly driving secondary stolon formation and glucose promoting ramet generation. This study improved our understanding of stolon patterning and bud development in the sympodial stolon of strawberries.

## 1. Introduction

**Figure 1:**
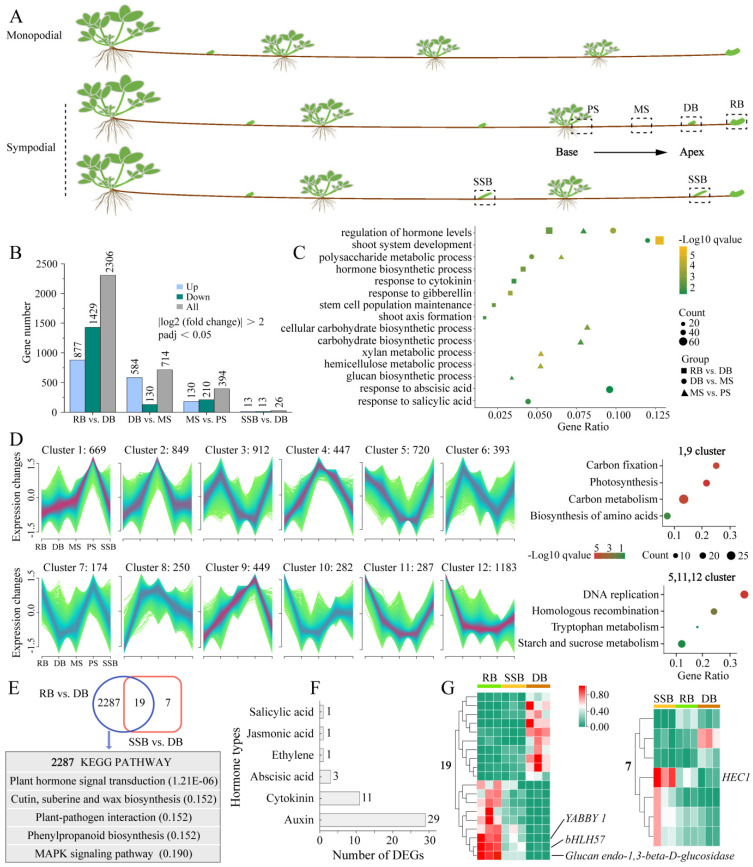
Transcriptome analyses of sympodial stolon. (**A**): Simplified schematic illustrating the two stolon patterns and sampling parts of sympodial stolon for RNA sequencing. The sampled parts include the proximal internode segment (PS), middle internode segment (MS), final dormant bud (DB), final ramet bud (RB), and secondary stolon bud (SSB). (**B**): Number of differentially expressed genes (DEGs) in contrasting groups, including comparisons of adjacent stolon parts and three types of buds. (**C**): GO enrichment of DEGs in adjacent stolon parts (Biological processes). The gene ratio is calculated as the number of genes annotated to a specific GO term divided by the total number of input genes, representing the gene abundance for that enriched GO term. (**D**): Mfuzz clustering analysis reveals dynamic changes in gene expression levels during stolon growth, classifying DEGs into 12 clusters. KEGG enrichment with clusters of two change patterns is also shown. (**E**): Venn diagram displaying DEGs among three types of buds, along with KEGG pathways of RB-specific DEGs; adjusted *p*-value of the pathway is provided in parentheses. (**F**): Types of hormones involved in plant hormone signaling transduction pathway and the number of each hormone-related DEGs. (**G**): Expression heatmap of shared 19 DEGs between RB vs. DB and SSB vs. DB (**left**). Expression heatmap of SSB-specific seven DEGs (**right**).

Strawberries are rosette plants with a shortened main stem, whorled by compound leaves [1]. Each leaf axil hosts an axillary bud, capable of developing into a flowering shoot for sexual reproduction, or forming a stolon/runner (a horizontal above-ground stem), on which ramets grow for vegetative propagation [2]. Propagating ramets through runners is the most effective method for raising strawberry seedlings [2]. Based on the growth pattern of ramets, strawberry stolons are classified into two types: monopodial and sympodial [3,4] (Figure 1A). The monopodial stolon features ramet development at each node after the initial single dormant bud, whereas the sympodial stolon alternates between forming a dormant bud at odd nodes and a ramet at even nodes [5,6]. However, the ramet spacing of sympodial stolon is wider than that of the monopodial type, leading to restricted seedling emergence per unit area. Therefore, the examination of pivotal genes and regulatory networks associated with the dormancy and outgrowth of strawberry stolon axillary buds is of great significance for the genetic improvement of strawberry stolons and augmenting land utilization efficiency.

The primary distinction between monopodial and sympodial stolons lies in whether the axillary buds at odd nodes remain dormant. This bud dormancy, a form of paradormancy, is controlled by biochemical signals from other organs or tissues and environmental conditions [7,8]. These biochemical signals include diverse hormones, carbohydrates, and other molecules [8,9], while the effects of environmental factors on bud outgrowth are typically mediated by hormones [10]. Different hormones often interact in synergistic, antagonistic, or additive manners to regulate axillary bud outgrowth [11]. For instance, auxin inhibits axillary bud outgrowth by inducing strigolactone and inhibiting cytokinin, while cytokinin promotes axillary bud outgrowth by facilitating auxin export, with strigolactone antagonizing cytokinin’s effect by inhibiting auxin export [9]. In addition to hormones, axillary bud dormancy or outgrowth is also regulated by carbohydrates/sugars [12]. In rose and pea, auxin inhibits axillary bud outgrowth by impeding cytokinin synthesis, while sugar promotes axillary bud outgrowth by enhancing cytokinin synthesis to counteract the effect of auxin [13,14]. Studies in *Arabidopsis thaliana* have demonstrated that sugar transporters regulate axillary bud development by modulating the expression of genes involved in hormone biosynthesis and signal transduction [15].

Previous studies on strawberry axillary buds have primarily focused on whether the buds on the main stem could develop into stolons, i.e., whether stolons were initiated or not. It was found that genes related to gibberellin synthesis and signal transduction, such as *GA20OX4* and *RGA1*, play a significant role in promoting strawberry stolon formation [2,16,17]. Additionally, the FaNAC2–FaHAN module may also be involved in controlling stolon formation through the regulation of gibberellin biosynthesis and auxin and cytokinin-responsive genes [18]. However, the molecular mechanisms regulating the formation of different stolon types after stolon initiation, particularly the alternating formation of dormant buds and ramet buds on sympodial stolons, remain unclear. A comparative study of sympodial stolons of *Fragaria vesca* and monopodial stolons of *F. pentaphylla* found that auxin and cytokinin antagonistically regulate the dormancy and outgrowth of axillary buds, potentially causing different types of strawberry stolon formation [5]. Further studies suggested that *FvYAB5.1* may play a pivotal role in the antagonistic relationship between cytokinin and auxin, resulting in the alternating growth of dormant buds and ramet buds on sympodial stolons [6]. Another study proposed that alternative splicing might be involved in different bud developments of cultivated strawberry sympodial stolons through the proteomics analysis of the dormant bud and ramet bud [19]. Nonetheless, these studies are conducted using allo-octoploid cultivated strawberries with a complex genetic background or buds from different species of stolons. The considerable genetic differences between species highlight the importance of examining different bud types within a single species. Sympodial stolon encompasses both dormant buds and ramet buds, and can represent the axillary bud development characteristics of monopodial and sympodial stolons simultaneously, rendering it suitable for the in-depth study of the formation and development mechanism of different strawberry stolon types.

The diploid *F. nilgerrensis* possesses sympodial stolons and is widely distributed in southwest China, renowned for its resistance to cold, drought, and diseases, as well as its white fruits with a unique peach-like aroma [20,21,22]. Its high-quality genome at the chromosome-level has been sequenced and made publicly available [21,23,24]. Furthermore, extensive research has been conducted on *F. nilgerrensis*, including population genomics [22,25] and epigenomics [26,27], aiming to position *F. nilgerrensis* as a new wild strawberry model species. Crucially, unlike the complex and large-scale genome of allo-octoploid cultivated strawberries, its genome is relatively small. Additionally, it has a short growth cycle and a high rate of gene conversion, making it an ideal material for studying the genetic mechanism regulating different types of strawberry stolon formation. In this study, we comprehensively analyze the sympodial stolons of *F. nilgerrensis* from the aspects of transcriptomics and phytohormone profiling. These analyses are supplemented by paraffin sections, exogenous hormone applications, and an in vitro sugar culture of dormant buds to elucidate the genetic and physiological mechanisms that control sympodial stolon formation. This study not only enhances our comprehension of the molecular mechanisms underlying sympodial stolon formation, laying a theoretical foundation for the future advancement of the strawberry propagation industry, but also provides clues for studying the stolon development mechanisms in other important crops.

## 2. Materials and Methods

### 2.1. Plant Material

In this study, plant tissues of *F. nilgerrensis* were collected from the meadows on Liangwang Mountain, Yunnan Province, China (24°45.474′ N, 102°54.910′ E) for RNA sequencing (RNA-seq), endogenous hormone level determination, and qRT-PCR analysis. The samples were collected at the end of June, during the rainy season (May to October, with 85% of the annual rainfall concentrated in this period), with average monthly temperatures ranging between 20 °C and 23 °C. Some individuals of *F. nilgerrensis* were transplanted into a greenhouse (20–26 °C, 55–68% relative humidity) at the Agricultural College of Yunnan University. Experiments involving paraffin sections, exogenous hormone treatments, and in vitro culturing of dormant buds were conducted using the newly emerged stolons of *F. nilgerrensis* plants grown in the greenhouse.

### 2.2. RNA Sequencing

To explore the dynamic changes of gene expression during sympodial stolon growth, we conducted RNA sequencing (RNA-seq) on five distinct parts (PS, MS, DB, RB, and SSB) of sympodial stolons in *F. nilgerrensis* (Figure 1A). Each of the five stolon parts was replicated three times and promptly flash-frozen using liquid nitrogen, then preserved at −80 °C for RNA-seq. The resulting clean reads from RNA-seq were then mapped to the reference genome of *F. nilgerrensis* using Hisat2 (version 2.0.5) [24]. Mapped read counts were normalized using Fragments Per Kilobase of transcript per Million mapped reads (FPKM). The prediction of novel transcripts was accomplished using StringTie (version 1.3.3b). DESeq2 (version 1.38.1) was used to analyze the differential expression of genes (DEGs) between comparisons [28]. Genes with |log2 (fold change)| > 2 and an adjusted *p*-value < 0.05 (*p*.adj < 0.05) were defined as DEGs. The Mfuzz (version 2.58.0) was applied for soft clustering analysis of gene expression changes during stolon development [29]. Gene Ontology (GO) enrichment was performed using the clusterProfiler (version 4.6.0) [30] Kyoto Encyclopedia of Genes and Genomes (KEGG) and enrichment was executed using the KOBAS online site (http://bioinfo.org/kobas (accessed on 16 June 2023)) [31].

### 2.3. Measurement of Endogenous Hormones

The hormone measurement samples were identical to those used for RNA-seq (PS, MS, DB, RB, and SSB), with each stolon part comprising three biological replicates. The samples were ground to powder in liquid nitrogen and then diluted in ultrapure water (dilution times: 1) with thorough vortexing. A 100 μL aliquot was homogenized with 400 μL of 50% acetonitrile containing mixed internal standards and extracted at 4 °C. The mixture was centrifuged at 12,000 rpm for 10 min. The supernatant (300 μL) passed through the HLB sorbent (first flow-through fraction) and then was eluted subsequently with 500 μL of acetonitrile (30%) (second flow-through fraction). These two fractions were combined, mixed well, and injected into the ultra-high performance liquid chromatography coupled to tandem mass spectrometry (UHPLC-MS/MS) system (ExionLC™ AD UHPLC-QTRAP 6500+, AB SCIEX Corp., Boston, MA, USA) for phytohormone quantitation. Separation was performed on a Waters XSelect HSS T3 column (2.1 × 150 mm, 2.5 μm) maintained at 45 °C. The mobile phase, consisting of 0.01% formic acid in water (solvent A) and 0.01% formic acid in acetonitrile (solvent B), was delivered at a flow rate of 0.3 mL/min. UHPLC-MS/MS utilized the stable isotope dilution technique, operating the mass spectrometer in multiple reaction monitoring modes. Parameters were as follows: IonSpray Voltage (Negative mode: −4500 V, Positive mode: 4500 V), Curtain Gas (35 psi), Ion Source Temp (550 °C), and Ion Source Gas 1 and 2 (60 psi). All phytohormone standards and stable isotope-labeled standards were obtained from ZZ Standards Co., Ltd. (Shanghai, China). Ultrapure water was purchased from Millipore (Burlington, MA, USA), and acetonitrile and formic acid were purchased from Thermo-Fisher Scientific (Fair Lawn, NJ, USA).

The comparative analysis of hormone content was conducted using the DESeq2 (version 1.38.1) with the criteria of |log2 (fold change)| > 1 and *p*.adj < 0.05 for detecting significant differences [28]. Correlation analysis between phytohormones and the 2287 DEGs specific to the RB was performed in Rstudio (version 4.2.1). The results were visualized using the ggplot2 (version 3.4.2). Subsequently, utilizing nine phytohormones as the trait file, Weighted Gene Co-Expression Network Analysis (WGCNA, version 1.71) was applied to all 28,165 genes. The parameter settings for WGCNA were as follows: networkType = Unsigned, Powers = 9, minModuleSize = 30, and MEDissThres = 0.2. Hub genes within the turquoise module were identified based on their Module Membership (MM, MM ≥ 0.8) and Gene Significance (GS, GS ≥ 0.8). The resulting co-expression network was visualized using Cytoscape (version 3.9.1).

### 2.4. Paraffin Section

Newly emerged stolons of *F. nilgerrensis* from the Agricultural College greenhouse were chosen for paraffin sectioning, using the same parts as those selected for RNA-seq and endogenous hormone measurement. Fresh samples (PS, MS, DB, RB, and SSB) were fixed in 50% formalin–acetic acid–alcohol (FAA) solution. Paraffin sectioning was conducted following the methodology outlined in a prior study [32]. The samples were sliced into 4 μm-thick sections using a Leica RM2255 microtome and mounted on Polysine Microscope Adhesion Slides. After staining with Safranin O-fast green, images of the sections were captured using the Leica Application Suite (LAS, version 3.8).

### 2.5. Exogenous Hormone Treatments

Newly emerged stolons of *F. nilgerrensis* were selected for hormone treatments, which consisted of two schemes. In the first scheme, dormant buds of sympodial stolons were treated with cytokinin (6-BA, 15 ppm), gibberellin (GA_3_, 15 ppm), and purified water (control group). Each treatment comprised three biological replicates, with bud numbers as follows: 24, 25, and 24 for 6-BA; 26, 25, and 25 for GA_3_; and 20, 20, and 20 for the control group. In the second scheme, dormant buds of sympodial stolons were subjected to different concentrations of cytokinin: 15 ppm (control group), 30 ppm, 50 ppm, and 100 ppm. Each treatment was replicated three times, with dormant bud numbers as follows: 47, 51, and 46 for 15 ppm; 50, 47, and 52 for 30 ppm; 52, 46, and 48 for 50 ppm; and 50, 50, and 51 for 100 ppm. In both schemes, dormant buds were sprayed with hormone daily at 17:00, continuing for 16 days.

### 2.6. Culturing Dormant Buds In Vitro

Newly emerged stolons of *F. nilgerrensis* were about 20 cm (5 to 6 weeks old), and stolon segments bearing dormant bud were harvested as explants. Explant sterilization followed a method previously used in *F. nilgerrensis* tissue culture [26]. Following thorough sterilization, the explants were transferred to culture medium with varying sugar conditions for growth. Each culture medium contained Murashige and Skoog (MS) without sugars and hormones, agar (7 g/L), and Plant Preservative Mixture (0.1%). These culture media were supplemented with different sugars, including sucrose, fructose, glucose, palatinose (a non-metabolizable sucrose analogue), mannose, and a blank control, with sugar concentrations set at 5 g/L. The pH of the culture medium was adjusted to 5.8. Each sugar culture underwent three replicates, with each replicate containing 50 segments bearing dormant buds. Subsequently, the explants were incubated with a 14 h light (25 ± 2 °C)/10 h dark (20 ± 2 °C) photoperiod, maintaining a light intensity of 38 μE m^−2^ s^−1^. Buds were imaged and recorded daily after culturing commenced.

### 2.7. qRT-PCR Analysis

We randomly selected 11 genes, either key genes identified by weighted gene co-expression network analysis or related to auxin homeostasis, for verification by qRT-PCR. High-quality RNA was extracted from the stolons of *F. nilgerrensis* using the CTAB method [33] and subsequently reverse-transcribed into cDNA using the PrimeScript RT kit (Takara, Kyoto, Japan). qRT-PCR was performed using the TB Green Premix Ex Taq II (Tli RNaseH Plus) kit (Takara, Kyoto, Japan) on a CFX96 Real-Time PCR Detection System with a C1000 Touch PCR (Bio-Rad, Hercules, CA, USA). Primers for each gene are listed in Supplementary Table S4. Relative gene expression was normalized using the 2^−ΔΔCt^ method, with *GADPH* as the internal reference [34]. Each qRT-PCR experiment included three independent biological replicates with three technical replicates.

## 3. Results and Discussion

### 3.1. Dynamic Changes of Differentially Expressed Genes (DEGs) during Sympodial Stolon Growth in F. nilgerrensis

To investigate the development mechanism underlying sympodial stolons, we collected newly emerged sympodial stolons of *F. nilgerrensis* for RNA sequencing (RNA-seq) and phytohormone measurements. Four distinct stolon parts spanning from the base to the apex of sympodial stolons were selected, encompassing two internodes and two types of buds. The internodes were situated between the second-to-last ramet and the final dormant bud, comprising the proximal internode segment (PS), adjacent to the ramet, and the middle internode segment (MS); the two types of buds were the final dormant bud (DB) and the final ramet bud (RB) (Figure 1A). Additionally, field observations found that axillary buds at odd nodes of sympodial stolons were typically dormant and enveloped by bracts; however, in certain natural microhabitats, these dormant buds broke dormancy and progressed into secondary stolons. Consequently, we also selected axillary buds that had just protruded from the bracts for RNA-seq, denoted as SSB (Figure 1A). With three replicates per part, 15 samples generated 99.14 Gb of clean data in RNA-seq (Supplementary Table S1). The clean reads were mapped to the *F. nilgerrensis* genome, with a unique mapping ratio exceeding 86.02% and Q30 scores ranging from 93.37% to 94.08%, indicating high data quality. All Pearson correlation coefficients (R2) among the replicates for each part were greater than 0.84 (Supplementary Figure S1A), and principal component analysis showed that the samples of PS, MS, and RB were grouped separately, while samples of SSB were scattered and mixed with DB (Supplementary Figure S1B). Axillary buds at odd nodes of sympodial stolons typically remain dormant, except in specific microhabitats where vegetation is sparse and the area is geographically open, allowing them to break dormancy and develop into secondary stolons. This suggests that environmental conditions can regulate the fate of axillary buds (DB or SSB) on stolons. The mixture of SSB and DB samples aligns with the natural growth pattern of sympodial stolons, further confirming the repeatability and reliability of the RNA-seq data.

The RNA-seq results revealed a progressive increase in the number of differentially expressed genes (DEGs) towards the apex of the sympodial stolon (Figure 1B). Specifically, we identified 394 DEGs between MS vs. PS, 714 DEGs between DB vs. MS, and 2306 DEGs between RB vs. DB. The Gene Ontology (GO) enrichment analysis of these DEGs revealed that the synthesis, response, and metabolism of various hormones were present across different stolon parts (Figure 1C), highlighting the crucial role of hormones in regulating the sympodial stolon development [5]. Notably, the carbohydrate biosynthetic process (GO:0016051), hemicellulose metabolic process (GO:0010410), and glucan biosynthetic process (GO:0009250) were uniquely enriched in MS vs. PS, suggesting that the internodes (MS, PS) of strawberry stolon may serve as carbohydrate reservoirs [35]. Additionally, to gain deeper insights into the dynamic changes of gene expression during sympodial stolon growth, we utilized Mfuzz for clustering DEGs with similar expression patterns, categorizing them into 12 clusters (Figure 1D). Clusters 1 and 9, comprising 1118 DEGs, displayed a continuous downregulation from the base to the apex of the sympodial stolon, with the lowest expression in the two active buds (RB, SSB). In contrast, clusters 5, 11, and 12, consisting of 2190 DEGs, showed an opposite expression pattern. The Kyoto Encyclopedia of Genes and Genomes (KEGG) enrichment analysis revealed that downregulated genes were enriched in a carbon fixation, carbon metabolism, etc., while upregulated genes were enriched in a starch and sucrose metabolism, underscoring the essential role of carbohydrates/sugars in sympodial stolon growth.

We also compared the three distinct buds (DB, SSB, and RB), using DB as the basis for comparison. A Venn plot showed that 2287 DEGs were specific to RB, 19 DEGs were shared between RB and SSB, and only seven DEGs were exclusive to SSB (Figure 1E). This suggests that the gene expression patterns of SSB and DB were very similar. Given the relatively large number of RB-specific DEGs, we performed KEGG enrichment with these genes. The results revealed that the plant hormone signal transduction pathway was the most significant (Figure 1E), encompassing 46 genes. These 46 genes were associated with six classes of hormones: auxin, cytokinin, abscisic acid, ethylene, jasmonic acid, and salicylic acid (Figure 1F). Among these, auxin had the most DEGs with 29, followed by cytokinin with 11, indicating the essential role of these two hormones in the formation of the ramet bud [5]. Moreover, among the 19 DEGs shared between RB and SSB, *YABBY1*, *Glucan Endo-1,3-beta-D-Glucosidase*, and *bHLH57* were the most prominent (Figure 1G). The absence of the *YABBY* in *Arabidopsis thaliana* has been shown to disrupt the auxin signaling network and lead to abnormal meristem development [36]. *Glucan Endo-1,3-beta-D-Glucosidase* and *bHLH57* were implicated in glucose release and trehalose synthesis [37,38,39]. Meanwhile, among the seven DEGs exclusive to SSB, *HEC1* (bHLH transcription factor) was the most prominent (Figure 1G). *HEC1* is intricately involved in the auxin and cytokinin-signaling network, modulating auxin biosynthesis and efflux by activating the expression of *YUC4*, *PIN1*, and *PIN3*, and influencing cytokinin action through the activation of type-A ARR genes in cucumber and Arabidopsis [40,41]. In summary, sympodial stolon development is closely tied to sugars and hormones, with auxin and cytokinin potentially serving as key hormones.

### 3.2. Endogenous Hormones Profile and Morphological Landscape of Sympodial Stolon in F. nilgerrensis, and Exogenous Hormone Treatment for Dormant Bud

**Figure 2:**
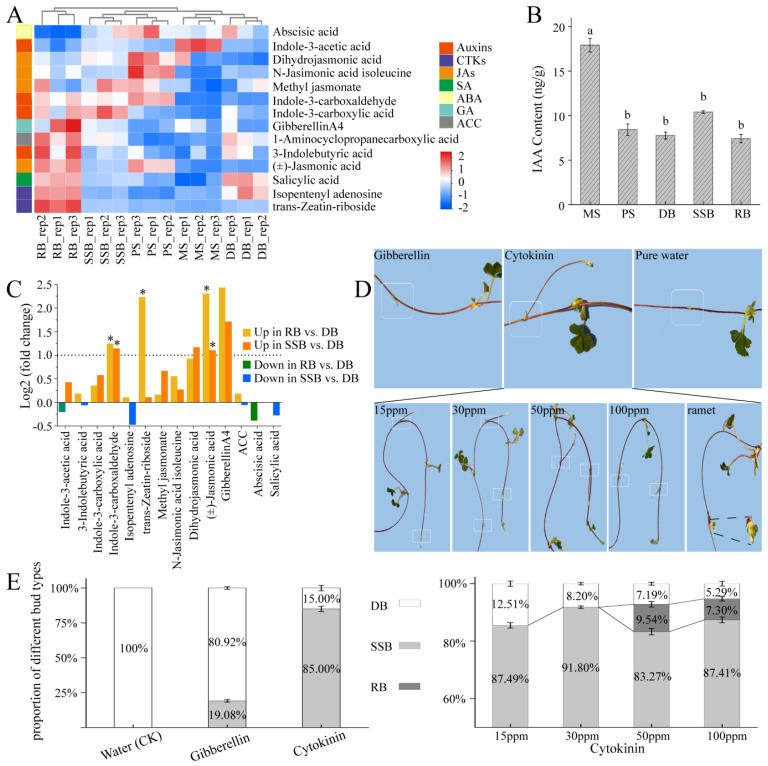
Endogenous hormone analyses of sympodial stolons and exogenous hormone treatment for dormant buds. (**A**): Heatmap illustrating the levels of 14 detected hormones in 5 stolon parts. The hormones, categorized into seven groups, are color-coded on the left: auxins, cytokinins (CKs), jasmonic acids (JAs), salicylic acid (SA), abscisic acid (ABA), gibberellin (GA), and 1-Aminocyclopropanecarboxylic acid (ACC), the precursor to ethylene. (**B**): Indole 3-acetic acid (IAA) content in the five stolon parts. Parts that do not share the same letter are significantly different (ANOVA, *p* < 0.05). (**C**): Differential analysis of hormone levels among the three buds. The dashed line represents the cut-off (|log2 (fold change)| = 1) for comparing hormone levels across the buds. Asterisks (“*”) indicate significantly differential hormones (|log2 (fold change)| > 1, *p*.adj < 0.05). (**D**,**E**): Exogenous hormone treatment for dormant buds of sympodial stolons in *Fragaria nilgerrensis* and the resulting proportion of different bud types after hormone treatment. Boxes around the nodes highlight the treated dormant buds. (i) Dormant buds were treated with 15 ppm cytokinin (6-BA), 15 ppm gibberellin (GA_3_), and purified water (control group). The number of dormant buds treated was 24, 25, and 24 for 6-BA; 26, 25, and 25 for GA_3_; and 20, 20, and 20 for purified water. Following the application of cytokinin and gibberellin, all dormancy-breaking buds developed into secondary stolon buds. (ii) Application of varying cytokinin concentrations: 15 ppm (control group), 30 ppm, 50 ppm, and 100 ppm. The number of dormant buds treated was 47, 51, and 46 for 15 ppm; 50, 47, and 52 for 30 ppm; 52, 46, and 48 for 50 ppm; and 50, 50, and 51 for 100 ppm. For the 15 ppm and 30 ppm treatments, all dormancy-breaking buds developed into secondary stolon buds. For the 50 ppm and 100 ppm treatments, the majority of dormancy-breaking buds developed into secondary stolon buds, while a small portion transitioned into ramet buds. The proportion of different bud types was calculated as the number of each bud type after hormone treatment divided by the total number of treated dormant buds. Data for each treatment were obtained from three biological replicates (mean ± SD). The numbers within the color blocks represent the proportions: white indicates non-dormancy-breaking buds (DB), gray represents buds that developed into secondary stolon buds (SSB), and black denotes buds that developed into ramet buds (RB) after dormancy-breaking.

To ascertain the key hormones influencing sympodial stolon development, we utilized the UHPLC-MS/MS method to measure endogenous hormones/hormone metabolites in the five parts of sympodial stolon (PS, MS, DB, RB, and SSB). The replicates for each part were consistently clustered together, distinct from replicates specific to other parts, underscoring the high repeatability and credibility of the measurements (Figure 2A). Fourteen hormones/hormone metabolites across seven categories were identified: indole-3-acetic acid (IAA) and its derivatives (Auxins), trans-Zeatin-riboside and isopentenyl adenosine (cytokinins, CKs), jasmonic acid and its derivatives (JAs), salicylic acid (SA), abscisic acid (ABA), gibberellinA4 (gibberellin, GA), and 1-Aminocyclopropanecarboxylic acid (ACC), the precursor to ethylene (Supplementary Table S2). The hormones/hormone metabolites content analysis showed that, except for IAA and ABA, RB exhibited a higher overall hormone level than other parts, suggesting heightened growth and metabolic activity in RB. Additionally, MS exhibited significantly higher levels of IAA compared to other stolon parts (Figure 2B, Supplementary Table S2). IAA is typically synthesized in the stem tip and young leaves [42]. As an internode of the sympodial stolon, MS likely benefits from polar auxin transport (PAT) mediated by auxin transporters [43,44], and the internodes may serve as auxin reservoirs [35].

Paraffin sectioning was performed across all five stolon parts. Tissue layers within the stolon, from exterior to interior, include the epidermis, thick cortex, and pith (Supplementary Figure S2). MS and PS, both internodes with near positions and similar sizes, had loosely expanded cortical cells, mostly spherosomes. In contrast, the three types of buds (DB, SSB, and RB) had similar sizes, but closely packed cortical cells, especially in RB. The cortex is recognized as a temporary storage tissue [19]. This cellular morphology supports the notion that internodes likely serve as reservoirs, as reported in *Alternanthera philoxeroides* [35]. Additionally, the new vascular bundles in the three bud types are inwardly connected with the vascular tissue of the primary stolon. Vascular bundles are responsible for transport [45], indicating that the developmental fate of buds is inevitably regulated by biochemical signals from the primary stolon, such as hormones.

Through a comparative analysis of hormones and their derivatives content, significant variations were observed in the levels of three compounds among the three buds (DB, SSB, and RB): indole-3-carboxaldehyde, trans-Zeatin-riboside, and jasmonic acid. Indole-3-carboxaldehyde, a hormone metabolite derived from the oxidative degradation of IAA, showed a significantly higher content in two active buds (RB, SSB) than in DB (Figure 2C). While the trans-Zeatin-riboside content in RB was notably higher than that in SSB and DB, indicating a positive role of cytokinin in ramet bud development [5]. Additionally, the level of jasmonic acid (JA) in RB was twice that of SSB, which, in turn, was twice that of DB. JA is known to enhance seedling growth and mitigate drought stress in crops such as wheat, soybean, and pearl millet [46,47,48]. Furthermore, JA levels and signaling increase in the buds of sorghum following leaf removal, and the application of JA promotes bud growth [49]. The differential distribution of JA among the three bud types may contribute to fostering active bud outgrowth in challenging environments. Interestingly, although gibberellin is essential for the formation and elongation of strawberry stolons [2,16], the differences in gibberellin levels did not exhibit significance among the three buds.

To corroborate these data analysis findings, we administered gibberellin and cytokinin to dormant buds of sympodial stolons in *F. nilgerrensis* (Figure 2D,E), using purified water as the control. The results indicated that no bud dormancy breaking was observed in the control group, and 19.08 ± 0.91% of the buds broke dormancy after treatment with 15 ppm gibberellin; however, the majority of these awakened buds ceased growth at approximately 3–5 cm. In contrast, buds treated with 15 ppm cytokinin exhibited a dormancy-breaking rate of 85.00 ± 5.00%, and all awakened buds developed into secondary stolons. Although none of the dormant buds treated with pure water broke dormancy under the controlled conditions in the greenhouse, previous field observations indicated that dormant buds occasionally break dormancy and develop into secondary stolons under natural and specific microhabitats. We speculate that these microhabitats may facilitate bud germination by influencing cytokinins level, as reported in *Rosa hybrida* [50,51]. To further understand cytokinin’s role, we treated dormant buds of sympodial stolons with varying concentrations: 15 ppm (control), 30 ppm, 50 ppm, and 100 ppm (Figure 2D,E). The dormancy-breaking rates for buds treated with 15 ppm and 30 ppm cytokinin were 87.49 ± 3.12% and 91.80 ± 0.45%, respectively, with all developing into secondary stolons’ post-dormancy release. After 50 ppm cytokinin treatment, 83.27 ± 3.72% of buds formed secondary stolons, while 9.54 ± 1.94% transitioned into ramets, resulting in an overall dormancy-breaking rate of 92.81 ± 2.40%. Under 100 ppm treatment, 87.41 ± 1.23% developed as secondary stolons, while 7.30 ± 1.22% became ramets, leading to an overall dormancy-breaking rate of 94.71 ± 1.12%. These results indicate that, with an increase in the cytokinin concentration, the dormancy-breaking rate of the buds increased slightly [5]. Furthermore, cytokinin primarily induces the development of secondary stolons, as the proportion of ramet formation was very low, less than 10%. This suggests the involvement of other roles, either acting coordinately with cytokinin or functioning independently, in the development of ramets.

### 3.3. Exploring Key Genes Responsible for Ramet Formation of Sympodial Stolons in F. nilgerrensis

**Figure 3:**
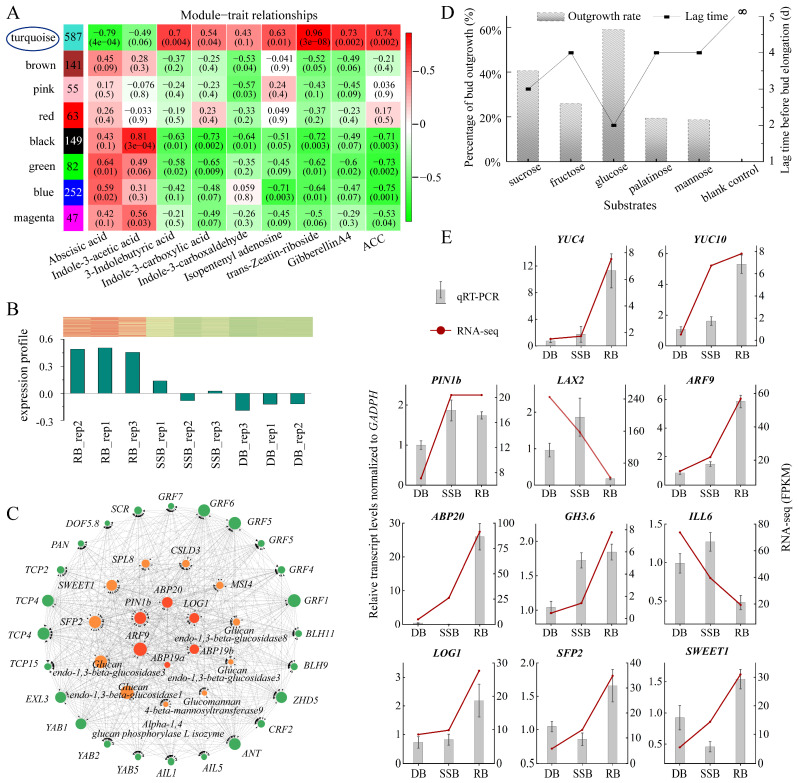
Weighted gene co-expression network analysis (WGCNA) unveils key genes involved in ramet bud formation and qRT-PCR validation of key genes. (**A**): Module–trait correlation. The numbers in the left column indicate the gene counts of each module. The heatmap displays correlations between modules (vertical axis) and phytohormones (horizontal axis), with *p*-values indicated in parentheses. (**B**): Expression profile of 362 hub genes within the turquoise module across the three bud types. The red–white–green heat map displays gene expression levels across the three bud types. (**C**): Key genes associated with ramet formation, categorized into three groups: crucial transcription factors (green pies on the outer circle), genes related to sugar (orange pies on the second circle), and genes related to auxin and cytokinin (bright red pies on the inner circle). (**D**): Cultivation of dormant buds under different sugar conditions. The bar chart illustrates the ratio of dormancy-breaking, and the line chart displays the time it takes. Data analysis is based on three biological replicates, with each replicate containing 50 dormant buds. (**E**): The expression levels of genes in the three types of buds were validated by qRT-PCR, using *GADPH* as an internal control, with gene expression levels in dormant buds (DB) normalized to a baseline of 1. The bar chart illustrates the standardized gene expression levels (means ± SD), while the line chart displays the expression levels determined by RNA sequencing. Data analysis is based on three biological replicates.

To identify additional roles specifically influencing ramet formation, we conducted a weighted gene co-expression network analysis (WGCNA) using all 28,165 genes. Prior to this, we performed a correlation analysis between the 2287 DEGs specific to the ramet bud (RB) and 14 endogenous hormones/hormone metabolites to determine suitable hormones/hormone metabolites for use as traits in WGCNA. The density plot revealed that trans-Zeatin-riboside had the largest correlation coefficient (0.91), while ABA had the smallest (−0.64) (Supplementary Figure S3B,C). Notably, the highest frequency correlation coefficient for jasmonic acids and its derivatives (JAs) and salicylic acids (SAs) were close to zero, suggesting a weak association with a ramet formation (Supplementary Figure S3D). Consequently, JAs and SAs were excluded, leaving nine hormones/hormone metabolites as traits. Employing WGCNA, the genes were grouped into eight modules. Among them, the turquoise module showed the strongest positive correlation with trans-Zeatin-riboside, 3-indolebutyric acid, isopentenyl adenine, gibberellin A4, and 1-Aminocyclopropanecarboxylic acid (ACC), and the strongest negative correlation with abscisic acid (ABA) and indole-3-acetic acid (Figure 3A). Furthermore, the expression profile of the 362 hub DEGs in the turquoise module showed that RB had the highest expression and DB had the lowest (Figure 3B). These findings suggest that the turquoise module likely encompasses key genes responsible for ramet formation.

Through the construction of the co-expression network within the turquoise module, we identified 41 genes falling into three categories: sugar-related genes, auxin and cytokinin-related genes, and transcription factors (TFs) (Figure 3C, Supplementary Table S3). Among the sugar-related genes, we identified two sugar transporters: *SWEET1* and *SFP2*. Sugar acquisition relies on transporters, with different transporters dedicated to different sugars [52]. SWEET1 acts as a glucose transporter, while SFP2 functions as a low-affinity transporter capable of transporting various monosaccharides, including glucose, fructose, and mannose [53,54]. To verify the function of sugars, we conducted an in vitro cultivation of dormant buds under various sugar conditions (Supplementary Figure S4). The tracking data showed that glucose took the shortest time to break dormancy, only 2 days, followed by sucrose, which took 3 days. Fructose, palatinose, and mannose required slightly longer, all 4 days, while no bud breaking dormancy was observed in the blank control group without any sugar during the 12-day observation period. At the same time, the dormancy-breaking rate of glucose was the highest, approximately 60.00%, followed by sucrose at 40.63% (Figure 3D). Fructose, palatinose, and mannose showed rates of 26.00%, 19.44%, and 18.68%, respectively. Intriguingly, all awakened buds transformed into ramets after dormancy disrupting, and the growth rate of buds under glucose treatment was the fastest (Supplementary Figure S5). These results demonstrate that sugar is indispensable for ramet formation, with glucose exhibiting higher effectiveness in promoting ramet formation.

Key genes related to auxin include efflux transporters (*PIN*) and response regulators (*ABP*, *ARF*). Auxin transport, response, inactivation, and synthesis coordinate regulated auxin homeostasis in plant tissue [55]. Auxin synthesis is primarily controlled by *YUCCA* (*YUC*) [56], while inactivation is predominantly directed by the *Gretchen Hagen3* family (*GH3s*), which encodes enzymes that catalyze the conversion of free IAA into conjugated forms [57]. The response is mediated by *ARF* and *ABP* [58], and efflux depends on the *PIN* family [59]. The expression levels of all these genes are higher in RB and SSB and lower in DB (Figure 3E, Supplementary Tables S5 and S6). In contrast, the auxin influx is facilitated by *AUX/LAX* [44], and the conjugated forms of IAA can be converted back into free IAA by enzymes encoded by the *ILR1/ILL* family [60]. These two types of genes (*AUX/LAX*, *ILR1/ILL*) exhibit the lowest expression in RB (Figure 3E). These data suggest that dynamic auxin homeostasis is established in vigorously growing buds (RB, SSB). Meanwhile, previous endogenous hormone measurements indicated that IAA abundance among the three buds (RB, SSB, and DB) shows no significant difference. Combining these observations, it is hypothesized that auxin homeostasis in axillary buds may impact auxin signaling to influence bud outgrowth and development independently of IAA levels. This result is preliminary, and further research is imperative to functionally define the role of auxin in regulating bud development.

**Figure 4:**
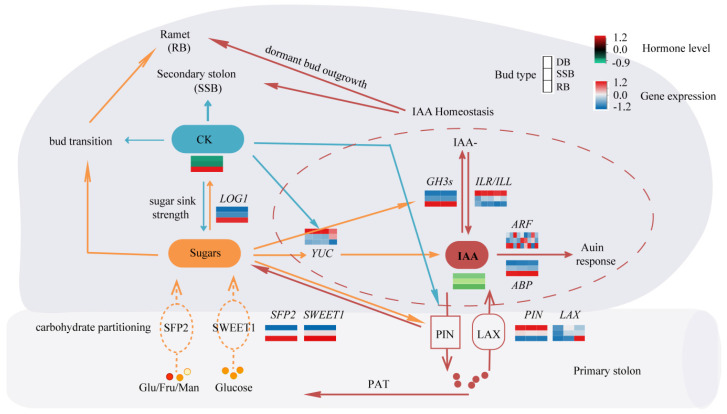
The speculated regulatory network of bud types. The thickness of lines signifies the strength of influence. The dashed lines indicate the possible transport functions of the two classes of sugar transporters. Light red–white–blue heat maps show gene expression levels in the three types of buds. The dark red–black–green heat map shows hormone levels in the three types of buds.

Bud development is a continuous and dynamic process, comprising dormancy breaking and development into distinct directions (ramet or secondary stolon). In vitro sugar culture of dormant buds promotes ramet formation, with reports suggesting that glucose and other sugars can modulate auxin homeostasis by influencing auxin synthesis, metabolism, and transport [61,62]. The key gene related to cytokinin is *LONELY GUY* (*LOG1*), which shows the highest expression in RB (Figure 3E, Supplementary Tables S5 and S6). *LOG1* encodes cytokinin riboside 5′-monophosphate phosphoribohydrolase, converting inactive cytokinin nucleotides to active free-base forms [63]. Elevated levels of glucose can induce cytokinin synthesis within the buds of pea and rose [13,14]. Therefore, it is hypothesized that glucose may trigger dormancy breaking by enhancing auxin homeostasis and cytokinin synthesis within the axillary buds of sympodial stolons, simultaneously provoking bud development into ramets. On the other hand, exogenous cytokinin application primarily induces the development of secondary stolons. While the distribution of various sugars among buds remains unchanged, increased cytokinin levels may enhance auxin transport and biosynthesis, as reported in Arabidopsis [58,64], facilitating auxin homeostasis to break bud dormancy and development into secondary stolons. Moreover, cytokinin application also leads to a small portion of dormant buds developing into ramets post-dormancy breaking, suggesting that heightened cytokinin levels may promote sugar accumulation, consistent with previous studies in rice [65,66]. Altogether, the interplay of cytokinin, auxin homeostasis, and glucose affects the axillary bud development of strawberry sympodial stolons (Figure 4).

## 4. Conclusions

Utilizing sympodial stolons of *F. nilgerrensis* as the materials, we uncovered the role of hormones and sugar in the axillary bud development of strawberry sympodial stolon through a comprehensive analysis integrating transcriptome and hormonal data. Cytokinin and glucose are likely key players in maintaining auxin homeostasis, thus influencing the axillary bud dormancy of stolon. Following bud dormancy breaking, cytokinin predominantly stimulates secondary stolon development, while glucose availability promotes ramet formation. Strawberries are high-value horticultural plants, with their commercial seedlings being the ramets propagated from stolons. Identifying how a cytokinin application stimulates dormant buds of sympodial stolons to develop into secondary stolons, which can also produce ramets, holds significant importance for improving seedling emergence rates per unit area. Our study has also narrowed down the range of genes regulating ramet development. However, the specific role of sugar transporters such as *SWEET1*, *SFP2*, and other key transcription factors requires further experimental verification. In summary, our work represents a preliminary exploration of sympodial stolon formation mechanisms, providing a reference for advancing the strawberry propagation industry.

## Data Availability

Supplemental information is available in the supporting information tab for this article online. The raw genomic reads generated in this study have been deposited in the NCBI Sequence Read Archive (BioProject PRJNA1050675).

## Supplementary Materials

The following supporting information can be downloaded at: https://www.mdpi.com/article/10.3390/plants13162241/s1, Figure S1: Analysis of RNA sequencing samples; Figure S2: Transection morphology of five stolon parts; Figure S3: Density plot illustrating correlation coefficients between 2287 differentially expressed genes (DEGs) specific to ramet buds and 14 phytohormones; Figure S4: In vitro cultivation of dormant buds from sympodial *F. nilgerrensis* under varied sugar conditions; Figure S5: Statistics on bud growth, with the average bud length on day 12 representing the outcome of each treatment; Table S1: Quality of RNA sequencing; Table S2: The content of 14 phytohormones (ng/g); Table S3: The categorization of the 41 key genes; Table S4: List of primer pairs used for qRT-PCR; Table S5: qRT-PCR validated the expression levels of genes in the three types of buds; Table S6: Comparison of gene expression levels among three types of buds.
