# Supplementary material for: Transcriptomic and Physiological Analyses for the Role of Hormones and Sugar in Axillary Bud Development of Wild Strawberry Stolon
Source: Plants (Basel). 2024 Aug 13;13(16):2241. doi: 10.3390/plants13162241 (PMC11359144; doi:10.3390/plants13162241)
Supplement: Supplementary file 1 [file plants-13-02241-s001.zip › Supplementary figure.pdf]

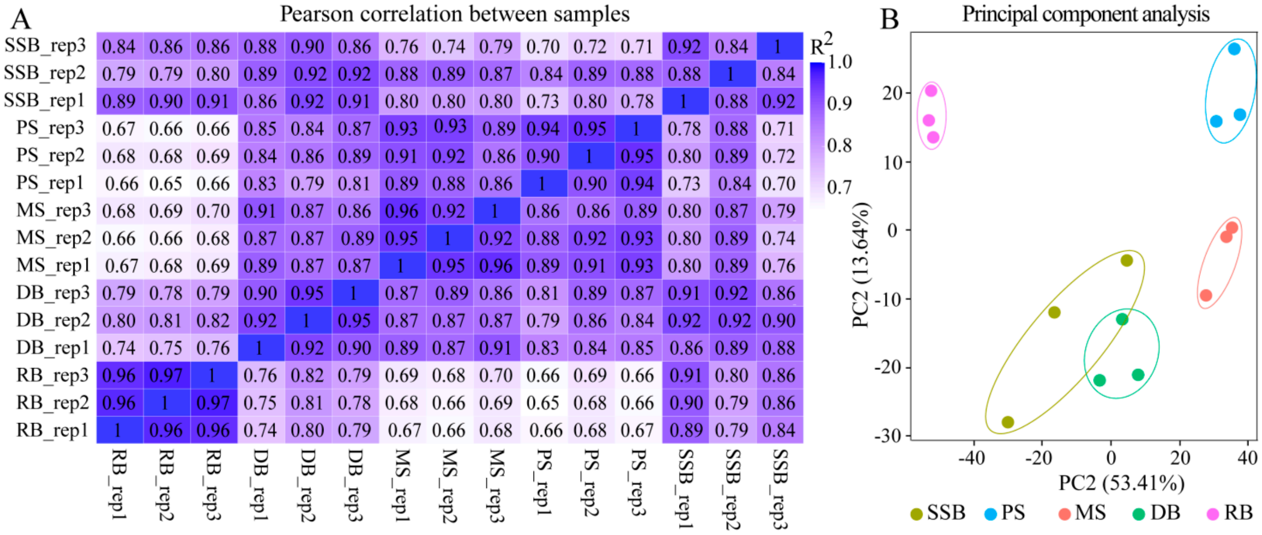

Figure S1. Analysis of RNA sequencing samples.

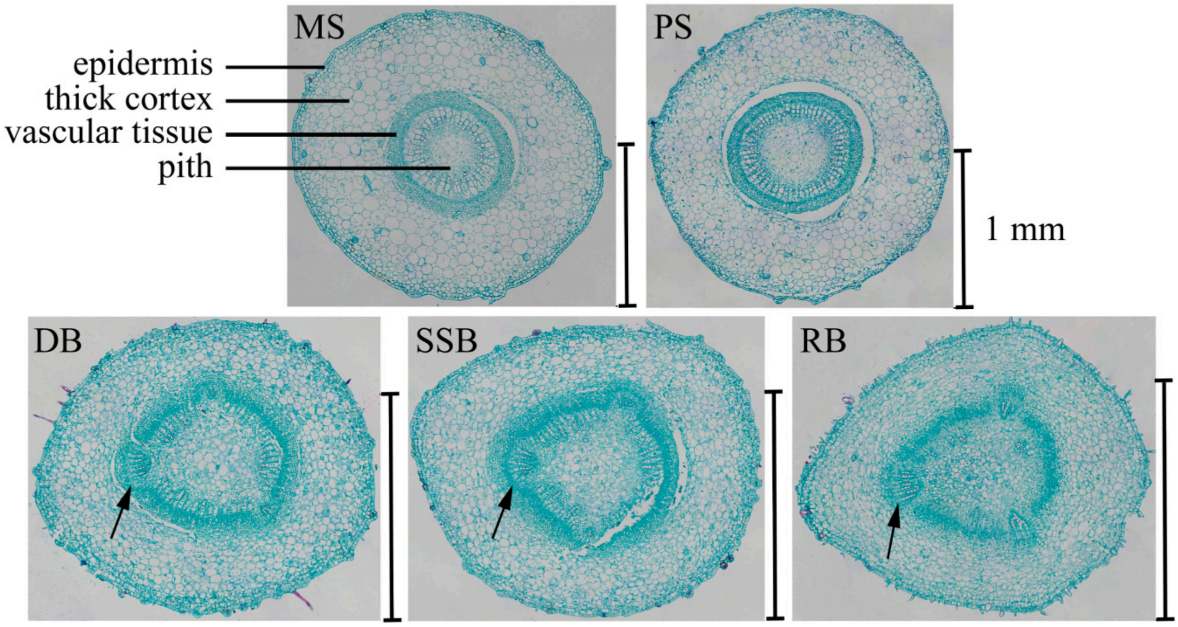

Figure S2. Transection morphology of five stolon parts. The upper images display the internode of the stolons at different positions (MS, PS), while the lower images exhibit the three types of buds (DB, NDB, RB), with arrows pointing to the new vascular tissue of the three bud types. The scale bar in all images represents 1 mm.

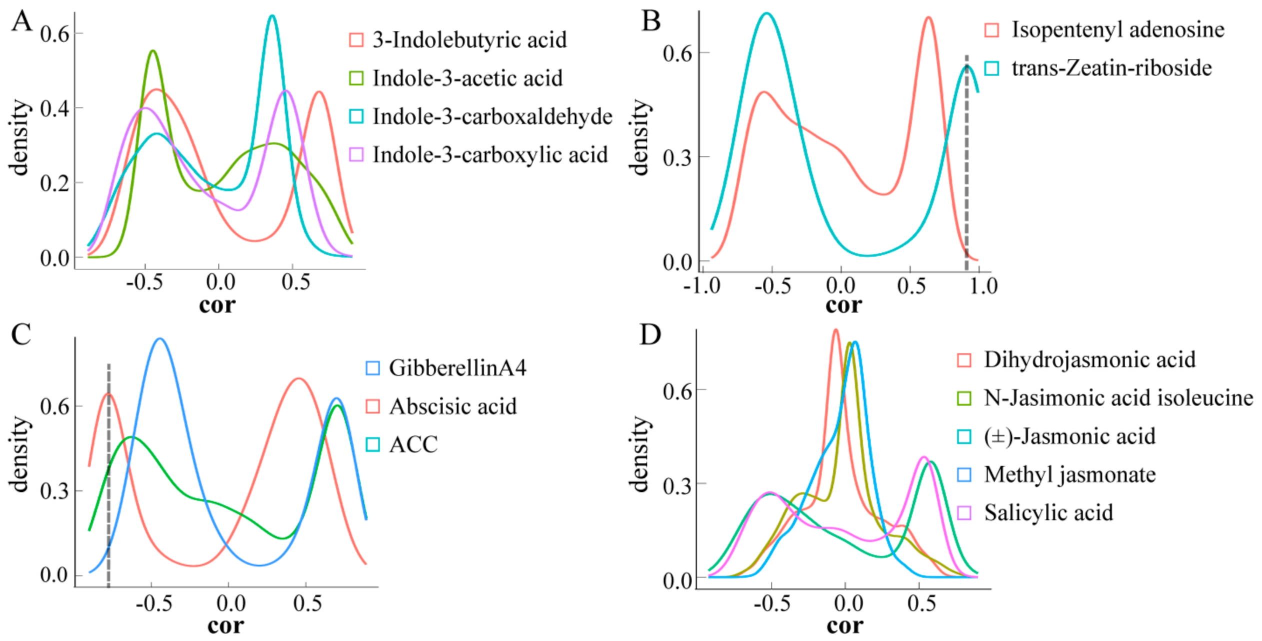

Figure S3. Density plot illustrating correlation coefficients between 2287 differentially expressed genes (DEGs) specific to ramet buds and 14 phytohormones. The X-axis value corresponding to the peak indicated the correlation coefficient with the highest frequency.

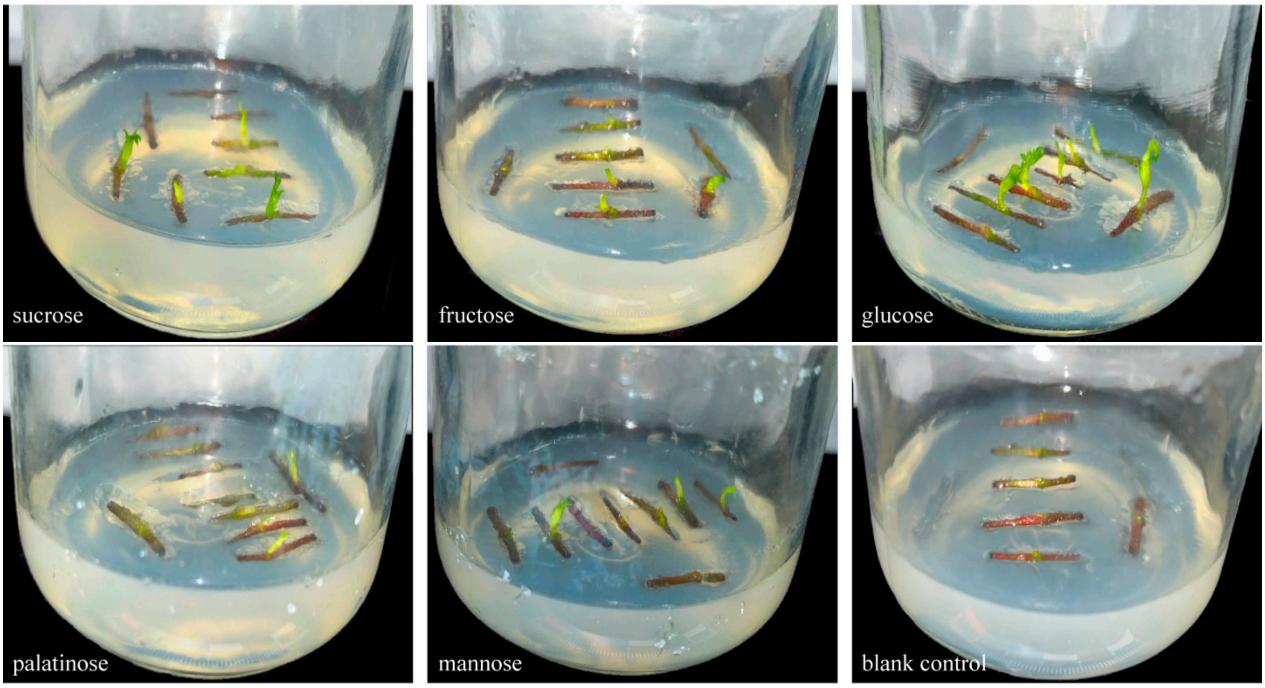

Figure S4. In vitro cultivation of dormant buds from sympodial *F. nilgerrensis* under varied sugar conditions.

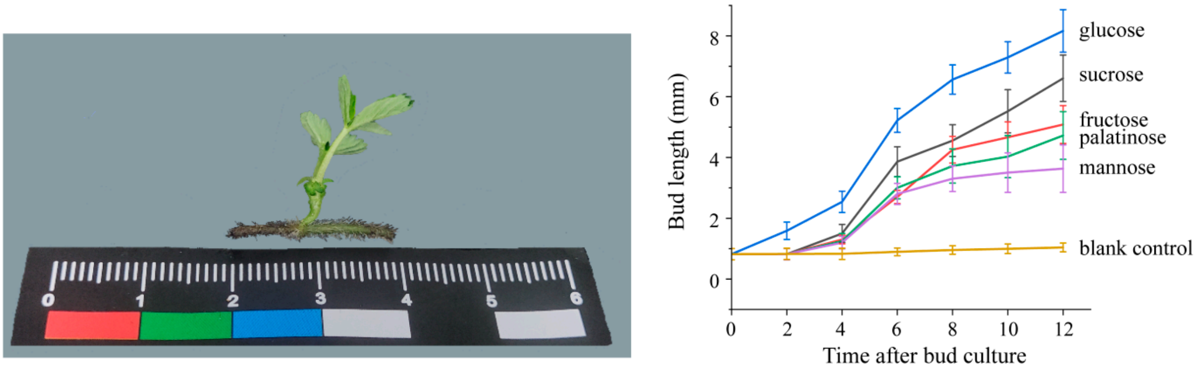

Figure S5. Statistics on bud growth, with the average bud length on day 12 representing the outcome of each treatment. Error bars represent the standard deviation from three biological replicates (means ± SD).
